# Aerobic exercise improves LPS-induced sepsis via regulating the Warburg effect in mice

**DOI:** 10.1038/s41598-021-97101-0

**Published:** 2021-09-07

**Authors:** Xishuai Wang, Zhiqing Wang, Donghui Tang

**Affiliations:** 1 Department of College of P.E and Sport, Beijing Normal University, No. 19, Xinjiekouwai St, Haidian District, Beijing, 100875 People’s Republic of China; 2 Department of Animal Genetic Resources, Institute of Animal Science, Chinese Academy of Agricultural Sciences, Beijing, 100193 People’s Republic of China

**Keywords:** Adaptive immunity, Immunology, Immunological disorders, Infectious diseases

## Abstract

We investigated the impact of aerobic exercise (AE) on multiple organ dysfunction syndrome (MODS), aortic injury, pathoglycemia, and death during sepsis. ICR mice were randomized into four groups: Control (Con), Lipopolysaccharide (LPS), Exercise (Ex), and Exercise + LPS (Ex + LPS) groups. Mice were trained with low-intensity for 4 weeks. LPS and Ex + LPS mice received 5 mg/kg LPS intraperitoneally for induction of sepsis. Histopathological micrographs showed the organ morphology and damage. This study examined the effects of AE on LPS-induced changes in systemic inflammation, pulmonary inflammation, lung permeability, and bronchoalveolar lavage fluid (BALF) cell count, oxidative stress-related indicators in the lung, blood glucose levels, plasma lactate levels, serum insulin levels, plasma high-mobility group box 1 (HMGB1) levels, glucose transporter 1 (Glut1) and HMGB1, silent information regulator 1 (Sirt-1), and nuclear factor erythroid 2-related factor 2 (Nrf-2) mRNA expression levels in lung tissue. AE improved sepsis-associated multiple organ dysfunction syndrome (MODS), aortic injury, hypoglycemia, and death. AE prominently decreased pulmonary inflammation, pulmonary edema, and modulated redox balance during sepsis. AE prominently decreased neutrophil content in organ. AE prominently downregulated CXCL-1, CXCL-8, IL-6, TNF-α, Glu1, and HMGB1 mRNA expression but activated IL-1RN, IL-10, Sirt-1, and Nrf-2 mRNA expression in the lung during sepsis. AE decreased the serum levels of lactate and HMGB1 but increased blood glucose levels and serum insulin levels during sepsis. A 4-week AE improves sepsis-associated MODS, aortic injury, pathoglycemia, and death. AE impairs LPS-induced lactate and HMGB1 release partly because AE increases serum insulin levels and decreases the levels of Glut1. AE is a novel therapeutic strategy for sepsis targeting aerobic glycolysis.

## Introduction

Sepsis, which has many complications, such as MODS, hypotension, and pathoglycemia, exhibits a high mortality rate stemming from a systemic infection^1–3^. Sepsis is characterized by an uncontrolled inflammatory response and oxidative stress^4,5^. Inflammatory mediators, including IL-1RN, IL-6, and TNF-α, and effector cells, including neutrophils and macrophages are important causes in the pathogenesis of sepsis^6,7^.

The exercise was known to treat many autoimmune and inflammatory diseases, such as chronic lung diseases and atherosclerosis because AE had immunomodulatory effects and modulated redox balance^8–10^. Previous researches demonstrated that LPS injection led to an excessive inflammatory response and oxidative stress injury and LPS injection generally accepted as somewhat modeling the septic condition^11,12^. Strikingly, limited research has demonstrated that exercise was a novel tool to prevent sepsis and its complications^11–14^. Exercised mice showed a survival benefit compared to unexercised mice in the septic model^15,16^. Exercise reduced acute lung injury (ALI) in mice subjected to LPS-induced sepsis^17^. Regular exercise reduced liver and kidney injury during severe polymicrobial sepsis^18^. MODS was a common complication of sepsis^19^. However, previous work ignored the impact of sepsis and exercise on the aorta. Besides, the underlying molecular mechanisms through which exercise improved sepsis-induced MODS have not yet been fully elucidated.

The ‘Warburg effect’, which was first found in cancer cells, was involved in innate and adaptive immunity^20,21^. Increasing evidence has demonstrated that activated immune cells, including macrophages, neutrophils, and T cells, switched from oxidative phosphorylation to aerobic glycolysis in a manner similar to tumor cells^20^. This alteration may contribute to the regulation of innate immune functions and represent a novel target for inflammatory diseases^21^. LPS injection induced a switch from oxidative phosphorylation to aerobic glycolysis in the immune cells including dendritic cells and macrophages^22^. Increased aerobic glycolysis consumed a large amount of glucose and produced a large amount of lactate. Increased serum lactate levels were a biomarker of mortality and organ failure during sepsis and that lactate clearance was a potential therapy for sepsis^23–25^. Besides, lactate can effectively stimulate macrophages to release HMGB1^26^. HMGB1 was involved in the development of the inflammatory response and was a promising therapeutic target for sepsis treatment^27,28^. The Warburg effect opened the door to developing new treatments for inflammatory diseases, including sepsis and ALI^21,29^. We attempted to demonstrate whether AE can impair LPS-induced lactate and HMGB1 release during sepsis.

## Materials and methods

### Animal

All protocols used in this study were approved by the Animal Experimental Welfare of the Institute of Animal Science, Chinese Academy of Agricultural Sciences (Beijing, China). All experiments were performed in accordance with the Animal Experimental Welfare of the Institute of Animal Science, Chinese Academy of Agricultural Sciences and the Guide for the Care and Use of Laboratory Animals published by the US National Institutes of Health. The authors have read the ARRIVE guidelines and the study was carried out in compliance with the ARRIVE guidelines. All mice were anesthetized via intraperitoneal injection of pentobarbital (0.2 mg/kg). The mice were euthanized with isoflurane.

### Experimental design and sepsis-induced protocol

The septic model was established by administering an intraperitoneal injection of 5 mg/kg LPS (O55:B5, Sigma-Aldrich, St. Louis, MO, USA). Forty mice were randomized into the following groups: (1) control group (Con), mice received a volume of normal saline equivalent to the volume of LPS; (2) LPS group (LPS), mice received 5 mg/kg LPS via intraperitoneal injection; (3) exercise group (Ex), mice were submitted to AE for 4 weeks; and (4) exercise plus LPS group (Ex + LPS), mice were submitted to AE for 4 weeks. Forty-eight hours later, mice received 5 mg/kg LPS via intraperitoneal injection. LPS were dissolved and diluted with 0.9% normal saline. The mice were killed at 6 h after LPS injection. Each group contained 10 mice.

### Exercise conditioning

Ex and Ex + LPS mice were submitted to aerobic exercise with the same treadmill protocol using a treadmill. After adaptive training for 3 days, the maximal exercise capacity test was measured, and Ex and Ex + LPS mice were trained in low-intensity exercise as previously described^30^. Treadmill aerobic training lasted for 8 weeks, once a day, and 60 min per session.

### Hemogram and BALF

Blood samples of mice were harvested and put into blood collection vessels. After centrifugation, the upper serum layer was harvested and frozen at − 20 °C immediately. The remaining lung tissues were frozen with liquid nitrogen. Two milliliters of ice-cold PBS was utilized for whole lung lavage, the whole BALF was flushed five times, and the output fluid was harvested. The supernatant was immediately stored at − 20 °C.

### Histopathology

The lung, heart, liver, kidney, and aorta tissue samples were dehydrated and embedded in paraffin. Five-µm-thick sections were stained with HE as previously described^31^. Photographs taken using an investigator (Olympus Corp., Japan) were used to perform morphometric measurements.

A semi-quantitative analysis of histopathologic acute injury was performed. The lung injury score, liver injury score, kidney injury score, and myocardial injury score were measured. The relevant content was provided in Supplementary Material 1.

### Detection of pulmonary permeability

To quantify the magnitude of pulmonary permeability, Wet dry weight ratio (W/D) was detected. Blotting papers were utilized to absorb liquid and blood on the surface of the lung tissues, and then the wet weight of the lung tissue was determined. The lung tissues were dried in a drying case until a stable dry weight was obtained. W/D = wet weight of the lung tissues/dry weight of the lung tissues.

### Cytokines

BALF levels of the proinflammatory cytokines (IL-6, CXCL-1, CXCL-8, and TNF-α) and anti-inflammatory cytokines (IL-10 and IL-1RN), and serum levels of IL-6, IL-10, and TNF-α were detected as previously described^30^.

### Detection of the Warburg effect

The blood glucose levels were detected using a blood glucose meter (Johnson Company). Lactate in serum was detected with a colorimetric l-lactate assay kit (Abcam, Cambridge, MA, USA). The serum insulin and HMGB1 levels were detected using Commercial kits (Shino Test Corporation, Tokyo, Japan). Besides, Glu1 and HMGB1 mRNA expression levels in the lung were detected.

### qRT-PCR

Total RNA which was extracted from lung tissue was used as a template for cDNA synthesis. We performed qRT-PCR as previously described^32^. GAPDH was served as the housekeeping gene. Supplementary Material 2 shows the murine PCR primer sequence information.

### Determination of oxidative stress index

MDA, MPO, GSH, and SOD expression was detected using spectrophotometry, as previously described following the instructions^33–36^.

### Assessment of survival rates

For survival analyses, 60 male mice (6 weeks old, 20–22 g) were randomly divided into three groups: (1) Con group, the mice received equal volumes of saline via intraperitoneal injection; (2) LPS group, the mice received 12 mg/kg LPS via intraperitoneal injection; (3) Ex + LPS group, the mice were trained for 4 weeks as previously described^30^. After the last training of 48 h, the mice received 12 mg/kg LPS via intraperitoneal injection. The number of dead mice was recorded every 6 h for 48 h. Each group included 20 mice.

### Data processing

We performed one-way ANOVA and graphed the figures using GraphPad Prism 9 software. The significance threshold was set to *P* < 0.05. All data are expressed as the mean ± SD (x ± s).

## Results

### Exercise regulated the Warburg effect during sepsis

**Figure 1:**
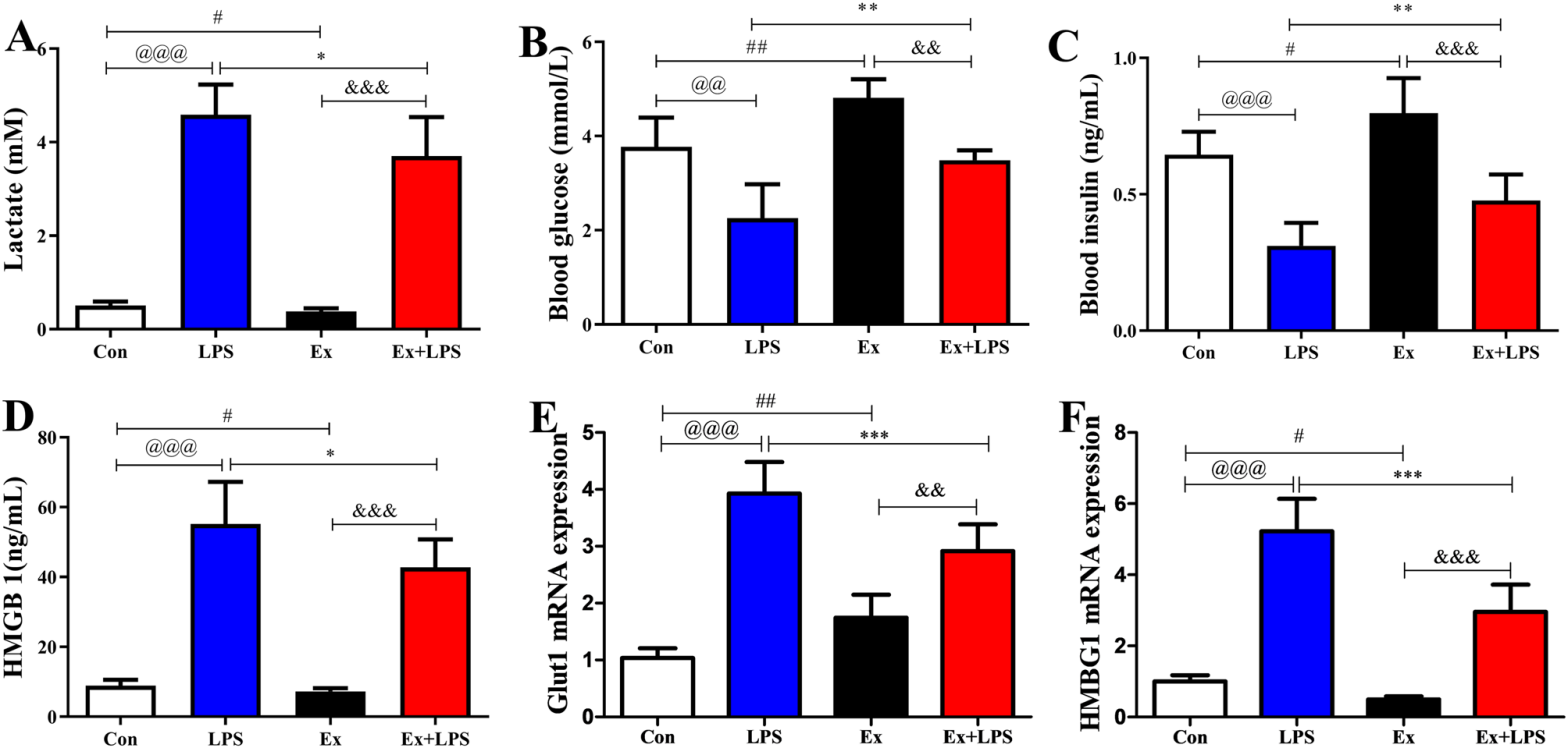
4 weeks of exercise pretreatment regulated the Warburg effect during sepsis. (**A**) Detection of serum lactate levels, (**B**) detection of blood glucose levels, (**C**) detection of serum insulin levels, (**D**) detection of serum HMGB1 levels, (**E**) detection of Glut1 mRNA expression levels in lung tissue, (**F**) detection of HMGB1 mRNA expression levels in lung tissue. ^@^*P* < 0.05, Con versus LPS groups; ^#^ *P* < 0.05, Con versus Ex groups; *P < 0.05, LPS versus Ex + LPS groups; ^&^*P* < 0.05, Ex versus Ex + LPS groups. *Glut1* glucose transporter 1, *HMGB1* High Mobility Group Box 1.

LPS injection markedly upregulated blood lactate levels (*P* < 0.001; Fig. 1A), serum HMGB1 levels (*P* < 0.001; Fig. 1D), and the mRNA expression levels of Glut (*P* < 0.001; Fig. 1E) and HMGB1 (*P* < 0.001; Fig. 1F) in lung tissue but markedly downregulated blood glucose levels (*P* < 0.001; Fig. 1B) and serum insulin levels (*P* < 0.001; Fig. 1C) compared to Con. A 4-week exercise pretreatment markedly upregulated serum insulin levels (*P* < 0.01) and blood glucose levels (*P* < 0.01) but markedly downregulated blood lactate levels (*P* < 0.05), serum HMGB1 levels (*P* < 0.05), and Glut (*P* < 0.001) and HMGB1 (*P* < 0.001) mRNA expression levels compared to LPS.

### AE prevented acute lung injury

**Figure 2:**
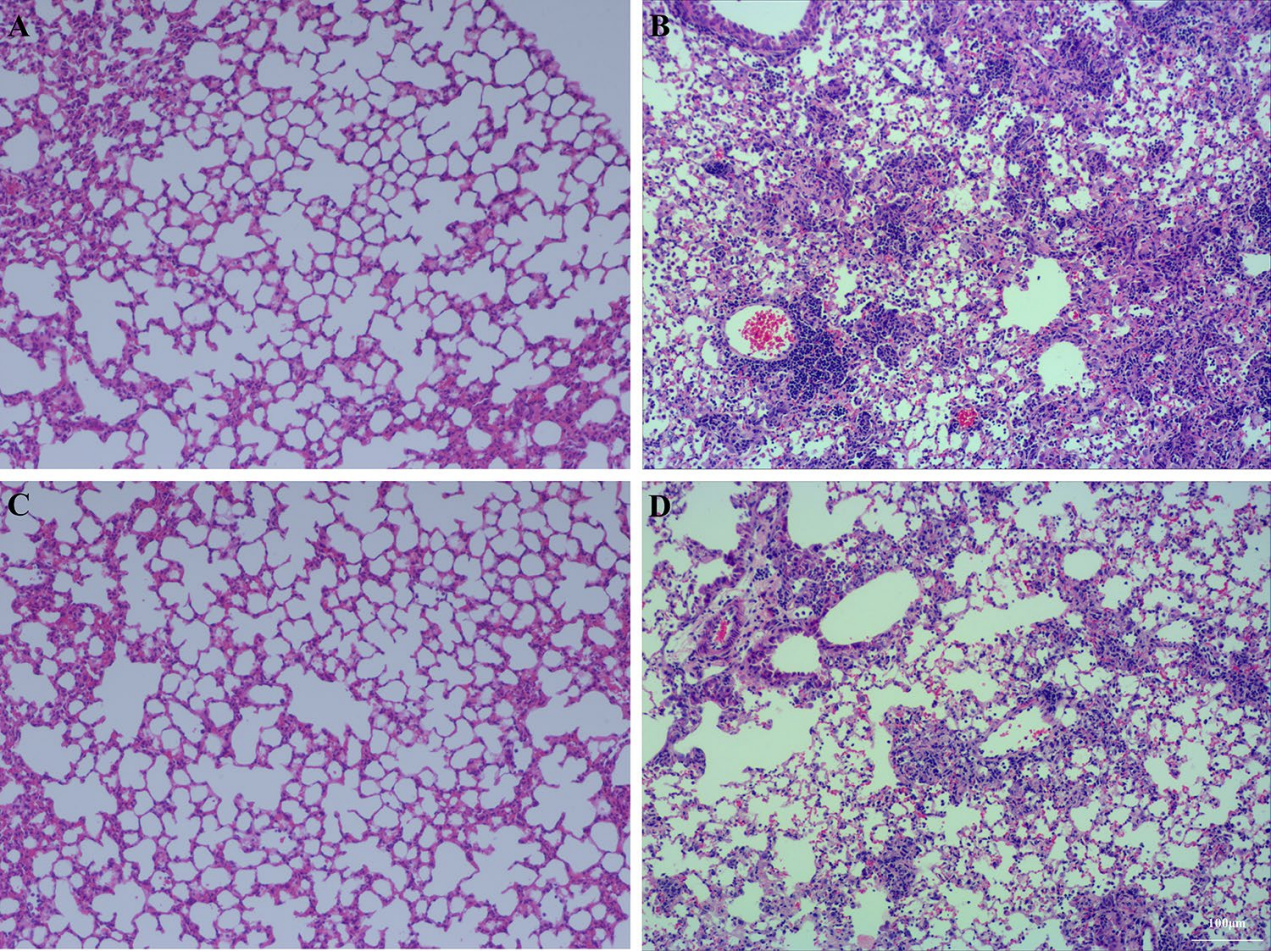
A photomicrograph of lung parenchyma used for morphological analysis. (**A**) Con group, (**B**) LPS group, (**C**) Ex group, (**D**) Ex + LPS group [(**A**–**D**) × 100 magnification].

Histologic assessment showed evidence of the degree of lung injury. In the Con group (Fig. 2A) and the Ex group (Fig. 2C), the lung tissues were intact and clear, the cells were neatly arranged, the intercellular substance was free of edema, and there were no symptoms of injury. LPS administration significantly increased lung injury, inflammatory infiltrates, and interstitial edema (Fig. 2B) compared to Con. AE significantly reduced the degree of lung injury, inflammatory cell infiltration, and interstitial edema (Fig. 2D). AE significantly decreased lung injury score compared to LPS (*P* < 0.001) (Supplementary Material 1).

### AE attenuated neutrophil content in lung tissue

**Figure 3:**
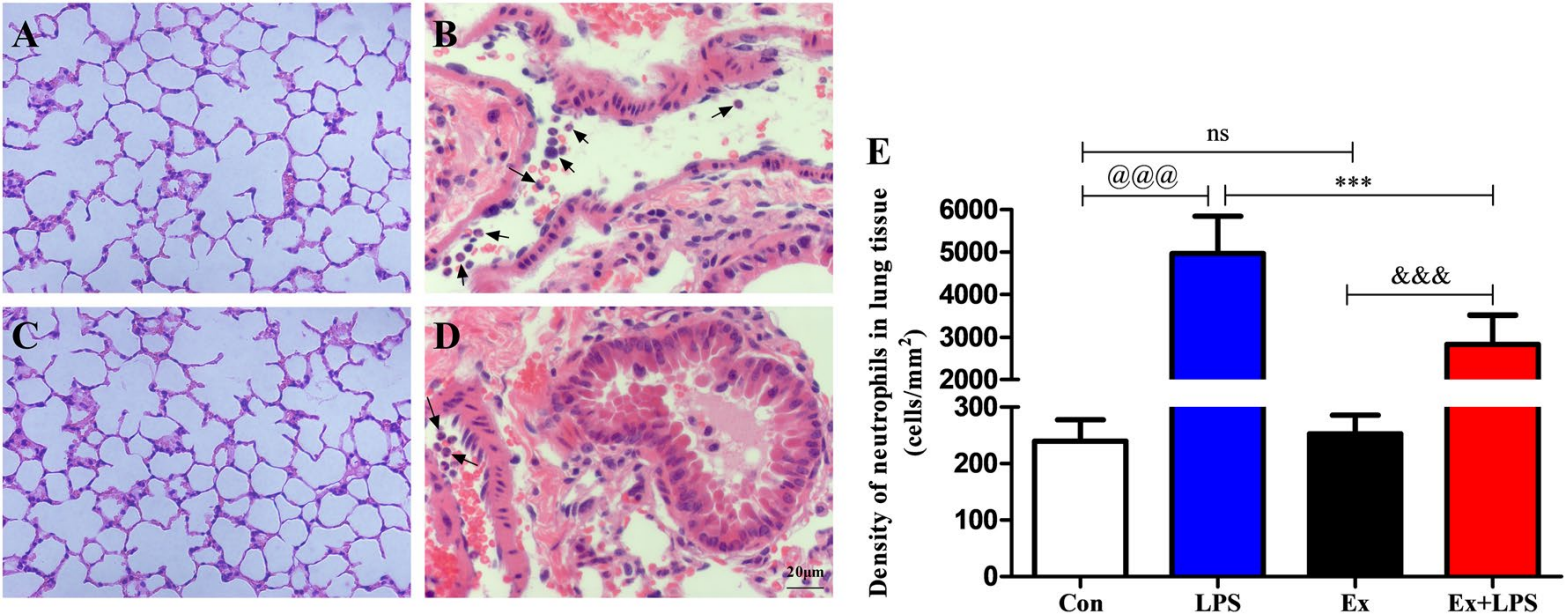
The density of neutrophils in lung tissue. (**A**) Con group, (**B**) LPS group, (**C**) Ex group, (**D**) Ex + LPS group, (**E**) detection of the density of neutrophils in lung tissue. Black arrows indicate neutrophils. ^@^*P* < 0.05, Con versus LPS groups; *P < 0.05, LPS versus Ex + LPS groups; ^&^*P* < 0.05, Ex versus Ex + LPS groups [(**A**–**D**) × 100 magnification].

Compared with the Con group (Fig. 3A) and the Ex group (Fig. 3C), there was a large amount of neutrophil infiltration after LPS administration (Fig. 3B). Compared with the LPS group, a 4-week exercise pretreatment prevented the upregulation of the neutrophil infiltration (Fig. 3D). The number of neutrophils increased significantly after the administration of LPS (*P* < 0.001). A 4-week exercise pretreatment prevented the upregulation of the number of neutrophils (*P* < 0.001) in mice with sepsis (Fig. 3E).

### Cell counts in BALF

LPS injection prominently increased the number of macrophages (*P* < 0.001) and neutrophils (*P* < 0.001) in BALF. A 4-week exercise pretreatment prevented the increase in the number of macrophages (*P* < 0.01) and neutrophils (*P* < 0.01) in mice with sepsis. LPS injection or AE administration did not change the number of lymphocytes and eosinophils in BALF (Table 1).

**Table 1:** Total and differential cell counts in BALF (cells/mL).

Group	Con	Ex	LPS	Ex + LPS	P
Total cells	1.33 ± 1.11	1.26 ± 1.41	10.88 ± 2.14^@@@^	8.58 ± 0.94*	< 0.01
Neutrophils	0.04 ± 0.02	0.03 ± 0.02	7.55 ± 1.23^@@@^	5.84 ± 2.38*	< 0.01
Lymphocytes	0.04 ± 0.03	0.05 ± 0.04	0.16 ± 0.11	0.14 ± 0.16	< 0.01
Macrophages	1.06 ± 0.48	0.96 ± 0.39	3.14 ± 0.60^@@@^	2.64 ± 0.19*	< 0.01
Eosinophils	0.02 ± 0.04	0.02 ± 0.03	0.01 ± 0.03	0.04 ± 0.05	< 0.01

The number of total cells, neutrophils, and macrophages, lymphocytes, and eosinophils in BALF was measured. ^@^*P* < 0.05, Con versus LPS groups. *P < 0.05, LPS versus Ex + LPS groups.

LPS injection prominently increased neutrophil content in the liver (*P* < 0.001), kidney (*P* < 0.001), and heart tissues (*P* < 0.001), while exercise prominently attenuated neutrophil content in the liver (*P* < 0.01), kidney (*P* < 0.01), and heart tissues (*P* < 0.01) during sepsis (Supplementary Material 1).

### BALF and serum cytokine levels

We detected a prominent effect of LPS administration in upregulating BALF levels of CXCL-1 (*P* < 0.001), CXCL-8 (*P* < 0.001), IL-6 (*P* < 0.001), IL-10 (*P* < 0.001), and TNF-α (*P* < 0.001) and downregulating BALF levels of IL-1RN (*P* < 0.001). We detected a prominent effect of AE administration downregulating BALF levels of CXCL-1 (*P* < 0.05), CXCL-8 (*P* < 0.01), IL-6 (*P* < 0.05), IL-10 (*P* < 0.01), and TNF-α (*P* < 0.001) and upregulating BALF levels of IL-10 (*P* < 0.05) and IL-1RN (*P* < 0.05) during sepsis (Table 2).

**Table 2:** Cytokine levels in BALF (pg/mL).

	Con	Ex	LPS	Ex + LPS	P
CXCL-1	9.99 ± 5.65	6.88 ± 4.25	594.82 ± 128.67^@@@^	466.94 ± 103.79*	< 0.01
CXCL-8	12.38 ± 3.54	7.62 ± 2.65	365.45 ± 82.66^@@@^	255.45 ± 76.51**	< 0.01
IL-6	8.68 ± 2.36	50.57 ± 15.47	1226.56 ± 97.69^@@@^	1064.44 ± 30.75*	< 0.01
IL-1RN	54.15 ± 3.88	47.86 ± 4.11	20.45 ± 10.79^@@@^	38.52 ± 15.17*	< 0.05
IL-10	9.56 ± 1.6	56.37 ± 38.01	495.83 ± 136.6^@@@^	660.74 ± 174.17**	< 0.01
TNF-α	18.34 ± 16.43	25.19 ± 14.45	343.28 ± 97.7^@@@^	251.64 ± 59.62***	< 0.01

The levels of CXCL-1, CXCL-8, IL-6, IL-1RN, IL-10, and TNF-α in BALF were detected. ^@@@^*P* < 0.05, Con versus LPS groups. *P < 0.05, LPS versus Ex + LPS groups. **P < 0.01, LPS verus Ex + LPS groups. ***P < 0.001, LPS verus Ex + LPS groups.

We detected an effect of LPS administration in upregulating the serum levels of IL-6 (*P* < 0.001), IL-10 (*P* < 0.001), and TNF-α (*P* < 0.001). The serum levels of TNF-α, IL-6, and IL-10 were no difference between the LPS and Ex + LPS groups (Table 3).

**Table 3:** Cytokine levels in serum (pg/mL).

	Con	Ex	LPS	Ex + LPS	P
IL-6	10 ± 5.65	6.88 ± 4.25	1419.98 ± 580.11^@@@^	1669.21 ± 216.26	< 0.01
IL-10	36.44 ± 32.23	50.57 ± 15.41	249.89 ± 97.68^@@@^	254.44 ± 110.75	< 0.01
TNF-α	14.15 ± 3.88	17.86 ± 4.11	130.45 ± 36.79^@@@^	138.51 ± 58.17	< 0.01

The levels of IL-6, IL-10, and TNF-α in serum were detected. ^@^*P* < 0.05, Con versus LPS groups.

### AE relieved liver injury

**Figure 4:**
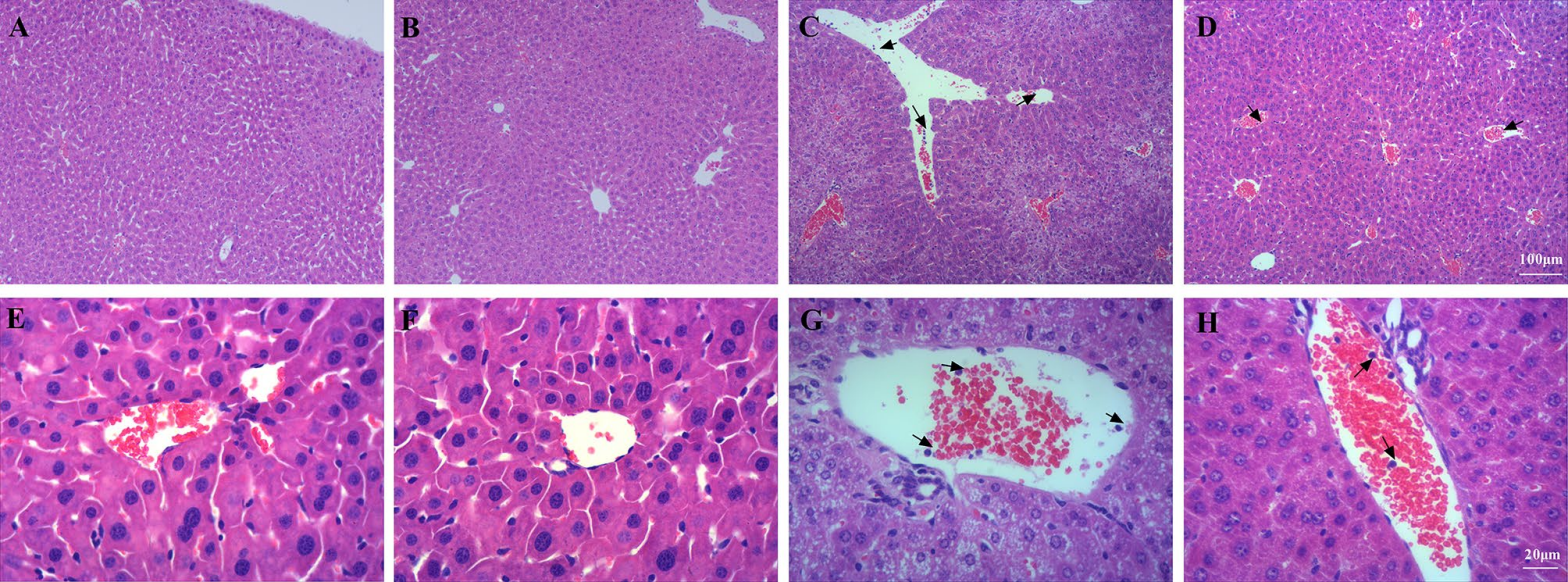
A photomicrograph of liver tissues used for morphological analysis. (**A**) Con group, (**B**) LPS group, (**C**) Ex group, (**D**) Ex + LPS group. White arrows indicate neutrophils [(**A**–**D)** × 100 magnification; (**E**–**H**) × 400 magnification].

The liver lobules of the mice in the Con group (Fig. 4A,E) and the Ex group (Fig. 4B,F) were intact and clear, the cells were neatly arranged, the intercellular substance was free of edema, the liver stripes were clear and regular, and there were no symptoms of injury. The liver lobules of the mice in the LPS group were severely damaged, the liver cells swelled, the intercellular substance disappeared, and there was a large amount of neutrophil infiltration (Fig. 4C,G). The liver lobules of the mice in the Ex + LPS group had significantly less liver tissue structural damage, with clearer liver lobules and a small amount of neutrophil infiltration (Fig. 4D,H).

LPS administration notably upregulated the markers of liver disease ALT (*P* < 0.001) and AST (*P* < 0.001) levels compared to Con. AE notably downregulated the ALT (*P* < 0.05) and AST (*P* < 0.05) levels compared to LPS. AE notably reduced the lung injury score compared to LPS (*P* < 0.001) (Table 4).

**Table 4:** Effects of AE on Gre, BUN, ALT and AST levels in serum.

Group	Con	Ex	LPS	Ex + LPS	P
Cre (μmol/L)	55.91 ± 11.98	61.3817.65	214 ± 10.35^@@@^	134 ± 15.49**	< 0.01
BUN (mmol/L)	7.99 ± 1.64	6.82 ± 1.93	13.56 ± 2.31^@@@^	10.59 ± 2.16*	< 0.01
ALT	27.65 ± 0.31	28.64 ± 0.39	48.55 ± 86^@@@^	37.2 ± 32.46**	< 0.01
AST	112.13 ± 15.9	108.97 ± 11.68	160.94 ± 49.6^@@@^	130.45 ± 26**	< 0.01

The markers of liver damage (ALT and AST) and markers of kidney damage (Cre and BUN) in serum were detected. ^@@@^*P* < 0.001, Con versus LPS groups. *P < 0.05, LPS versus Ex + LPS groups. **P <  0.01, LPS verus Ex + LPS groups.

### AE relieved kidney injury

**Figure 5:**
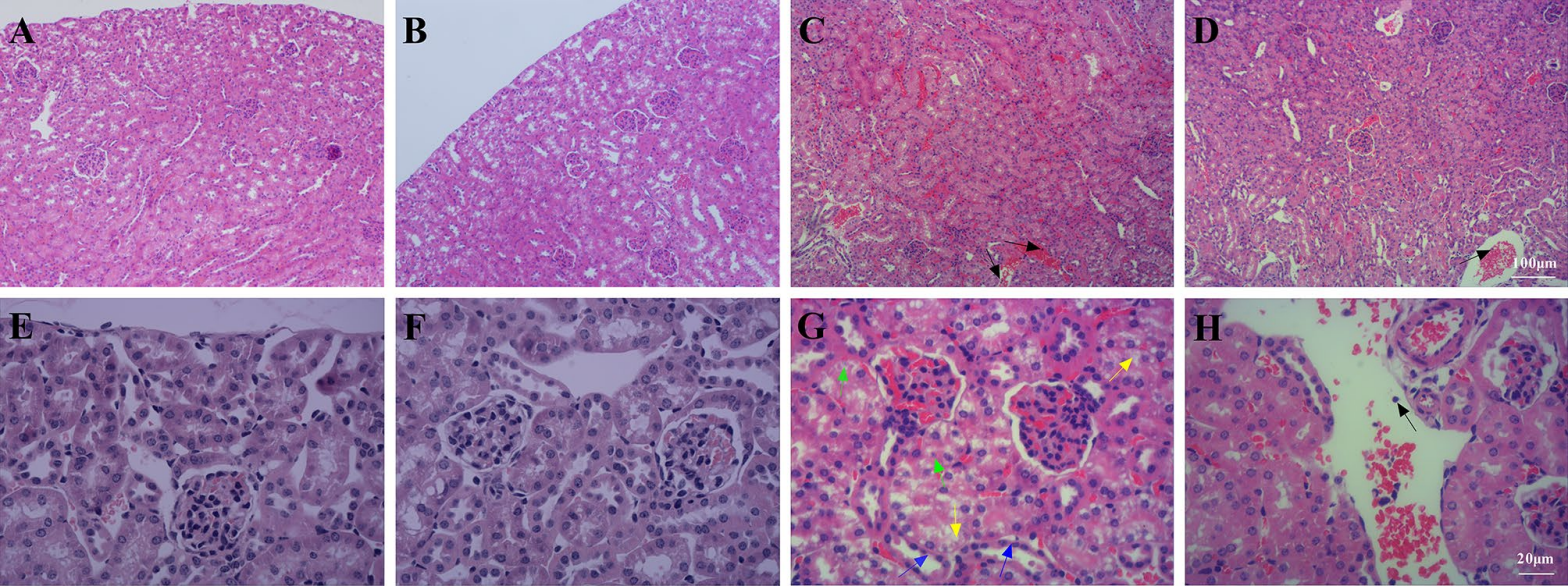
A photomicrograph of kidney tissues used for morphological analysis. (**A**) Con group, (**B**) LPS group, (**C**) Ex group, (**D**) Ex + LPS group. Black arrows indicate neutrophils. Yellow arrows indicate severe epithelial vacuolization. Blue arrows indicate flattening of the tubular epithelium. Green arrows indicate the appearance of an atypical shape with almost no nuclei [(**A**–**D**) × 100 magnification; (**E**–**H**) × 400 magnification].

The kidney tissues of the mice in the Con group (Fig. 5A,E) and in the Ex group (Fig. 5B,F) were intact and clear, the cells were neatly arranged, the intercellular substance was free of edema, and there were no symptoms of injury. Cortical tubular epithelial cells were well-shaped, and almost every epithelial cell owned intact nuclei. The kidneys of the mice in the LPS group were severely damaged, the cells swelled, and the intercellular substance disappeared, accompanied by a large amount of neutrophil (black arrows) and hemocyte infiltration, severe epithelial vacuolization (yellow arrows), flattening of the tubular epithelium (blue arrows), and the appearance of an atypical shape with almost no nuclei (green arrow) (Fig. 5C,G). The kidneys of the mice in the Ex + LPS group had significantly less kidney tissue structural damage than those of the LPS group, with clearer nephrons and a small amount of inflammatory cell infiltration, less flattening of tubular epithelium, and less vacuolization than those of septic mice, and the degree of damage was significantly reduced (Fig. 5D,H).

Compared with the Con group, LPS administration notably increased the levels of markers of kidney injury Cre (*P* < 0.001) and BUN (*P* < 0.001), while Cre (*P* < 0.05) and BUN (*P* < 0.05) levels were notably decreased in the Ex + LPS group compared with those in the LPS group. Compared with the LPS group, AE notably reduced the liver injury score (*P* < 0.001) (Table 4).

### AE prevented septicemic cardiomyopathy

**Figure 6:**
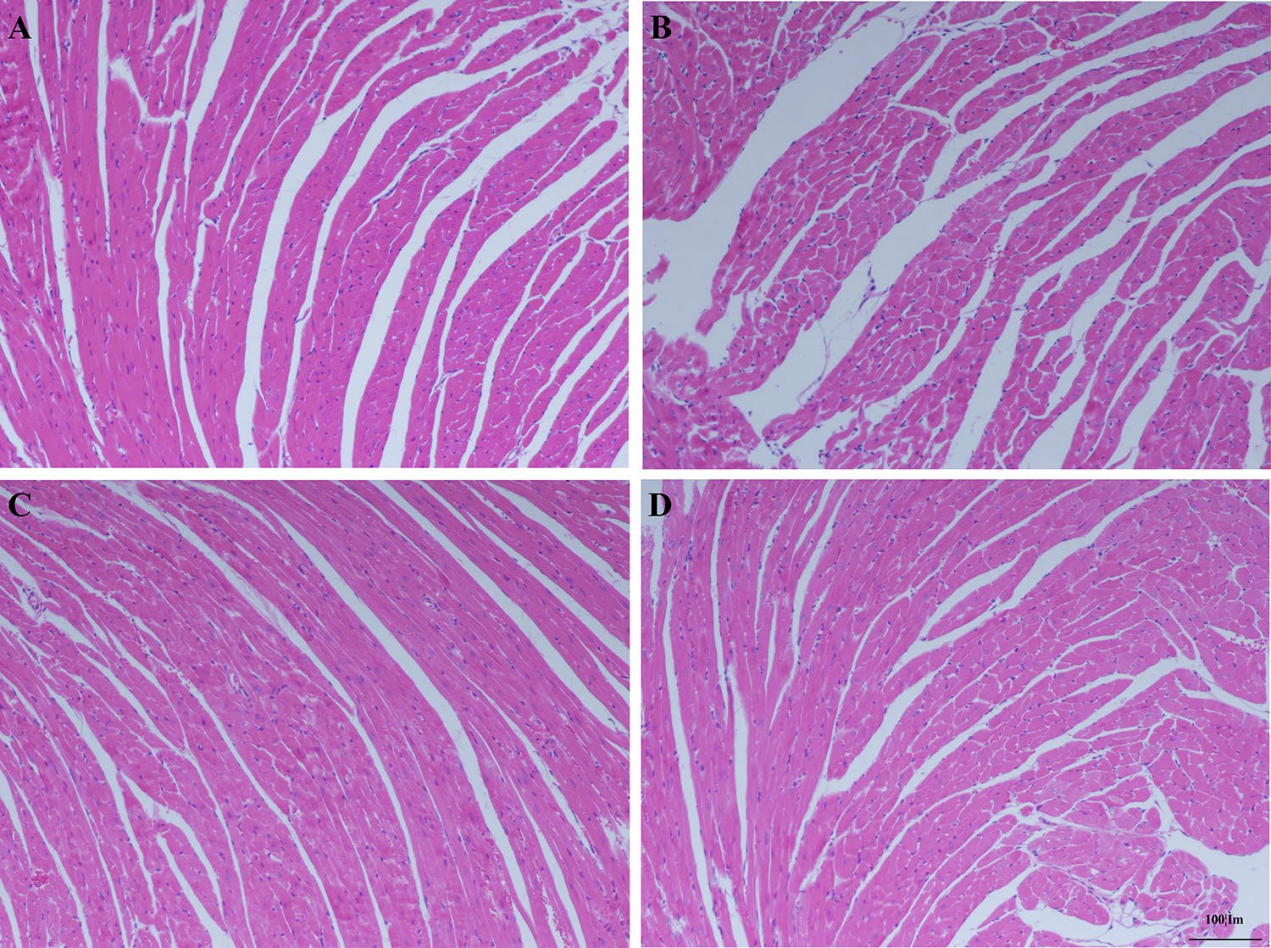
A photomicrograph of heart tissues used for morphological analysis. (**A**) Con group, (**B**) LPS group, (**C**) Ex group, (**D**) Ex + LPS group [(**A**–**D**) × 100 magnification].

In the Con group (Fig. 6A) and the Ex group (Fig. 6C), the myocardial tissue was uniformly stained, the myocardial fibers were arranged regularly and the interstitial spaces were normal. In the LPS group, myocardial tissues were disordered, myocardial degeneration occurred, dissolution occurred, and a large number of inflammatory cells infiltrated the muscle space (Fig. 6B). The inflammatory cells in the Ex + LPS group were less infiltrated, and the myocardial fiber tissue structure was normal. The distribution of muscle fibers was improved, but it did not completely return to the normal form (Fig. 6D). AE notably reduced the myocardial injury score (*P* < 0.001) compared to LPS (Supplementary Material 1).

### AE prevented aortic injury

**Figure 7:**
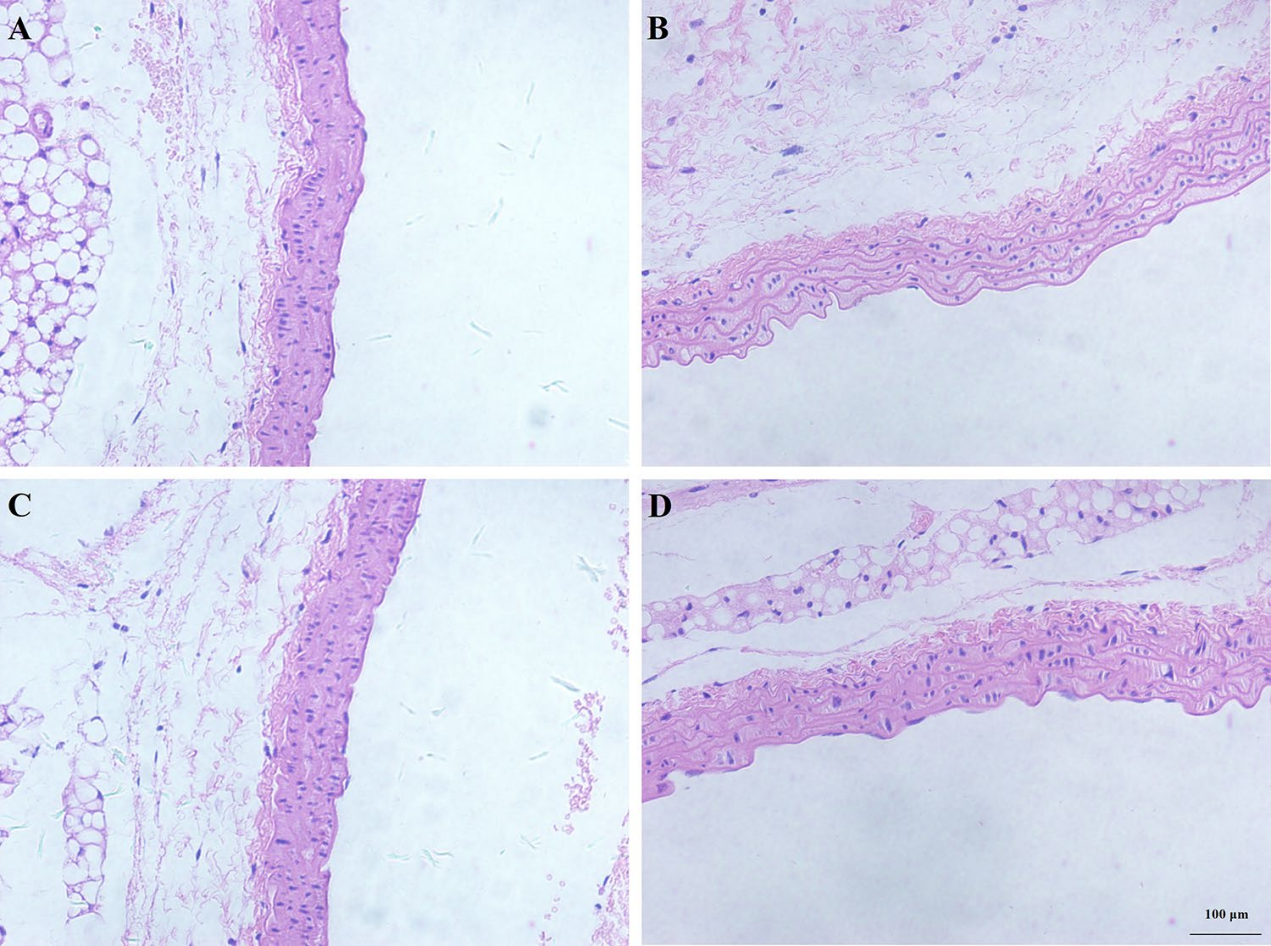
A photomicrograph of aortic tissues used for morphological analysis. (**A**) Con group, (**B**) LPS group, (**C**) Ex group, (**D**) Ex + LPS group [(**A**–**D**) × 100 magnification].

In the Con group (Fig. 7A) and the Ex group (Fig. 7C), the aorta was uniformly stained and arranged regularly, the endothelium was smooth and orderly, and the elastic fibers had a regular wavy-like shape. After LPS administration, the endothelium was not smooth and regular, the elastic fibers of the media became sparse, elastic fibers lost a regular wavy-like shape, and LPS administration significantly increased the aortic media thickness and decreased the area ratio of elastic fibers (Fig. 7B). AE notably increased the area ratio of elastic fibers and increased the aorta media thickness during sepsis (Fig. 7D).

LPS prominently increased the medial thickness of the aorta (*P* < 0.001) and prominently decreased the medium membrane elastic fiber area ratio (*P* < 0.001), which was reserved by a 4-week AE (Supplementary Material 1).

### AE prevented oxidative stress injury in lung tissue

**Figure 8:**
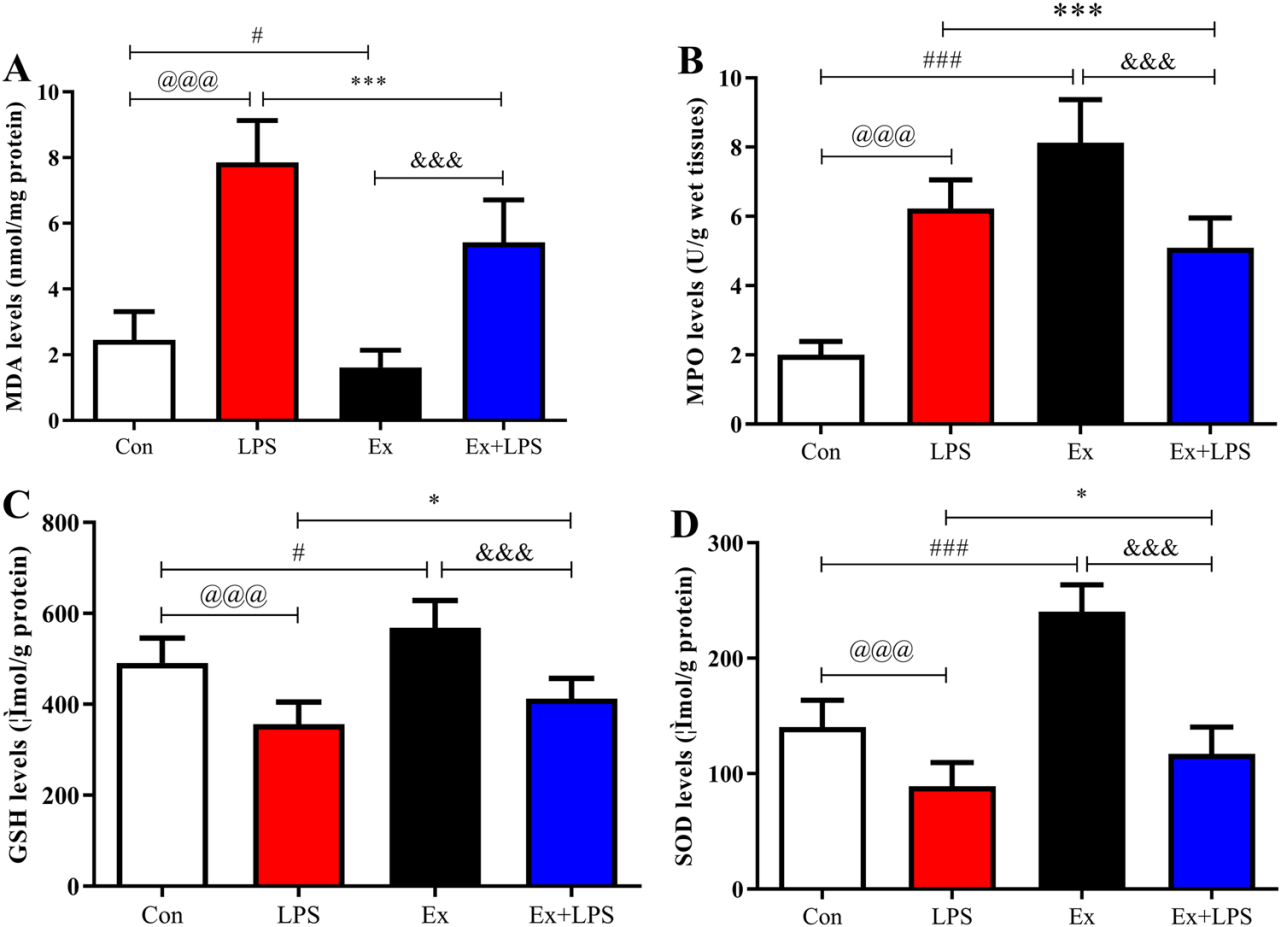
Detection of oxidative stress injury in lung tissue. The levels of MDA (**A**), MPO (**B**), GSH (**C**), and SOD (**D**) were detected. ^@^*P* < 0.05, Con versus LPS groups; ^#^*P* < 0.05, Con versus Ex groups; *P < 0.05, LPS versus Ex + LPS groups; ^&^*P* < 0.05, Ex versus Ex + LPS groups. *MDA* malondialdehyde, *MPO* myeloperoxidase, *GSH* glutathione, *SOD* superoxide dismutase.

LPS injection prominently upregulated MDA (Fig. 8A; *P* < 0.001) and MPO (Fig. 8B; *P* < 0.001) expression but prominently downregulated GSH (Fig. 8C; *P* < 0.001) and SOD (Fig. 8D; *P* < 0.001) expression compared to Con. AE prominently downregulated MDA (*P* < 0.001) and MPO (*P* < 0.001) expression but prominently upregulated SOD (*P* < 0.05) and GSH (*P* < 0.05) expression in lung tissue of septic mice.

### AE prevented lung injury via activating the Sirt-1/Nrf-2 pathway

**Figure 9:**
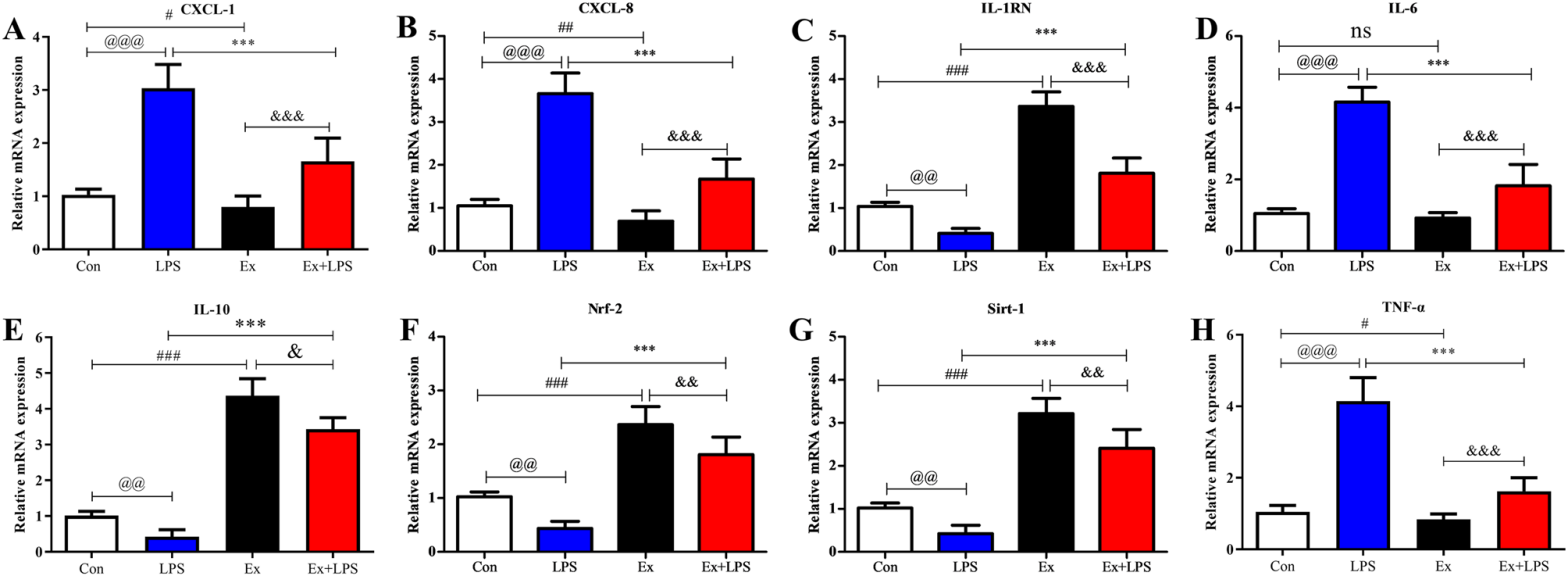
Detection of gene expression levels in lung tissues. The gene expression levels of CXCL-1 (**A**), CXCL-8 (**B**), IL-1RN (**C**), IL-6 (**D**), IL-10 (**E**), Nrf-2 (**F**), Sirt-1 (**G**), and TNF-α (**H**) were detected. ^@^*P* < 0.05, Con versus LPS groups; ^#^*P* < 0.05, Con versus Ex groups; *P < 0.05, LPS versus Ex + LPS groups; ^&^*P* < 0.05, Ex versus Ex + LPS groups.

LPS injection prominently upregulated the gene expression levels of CXCL-1 (Fig. 9A; *P* < 0.001), CXCL-8 (Fig. 9B; P < 0.001), IL-6 (Fig. 9D; *P* < 0.001), and TNF-α (Fig. 9H; *P* < 0.001) but prominently downregulated the gene expression levels of IL-1RN (Fig. 9C; *P* < 0.01), IL-10 (Fig. 9E; *P* < 0.01), Nrf-2 (Fig. 9F; *P* < 0.01), and Sirt-1 (Fig. 9G; *P* < 0.01) compared to Con. AE prominently upregulated the gene expression levels of IL-1RN (*P* < 0.001) and IL-10 (*P* < 0.001), Nrf-2 (*P* < 0.001), and Sirt-1 (*P* < 0.001) but prominently downregulated the gene expression levels of CXCL-1 (*P* < 0.001), CXCL-8 (*P* < 0.001), IL-6 (*P* < 0.001), and TNF-α (*P* < 0.001) in lung tissue.

### AE improved mortality

**Figure 10:**
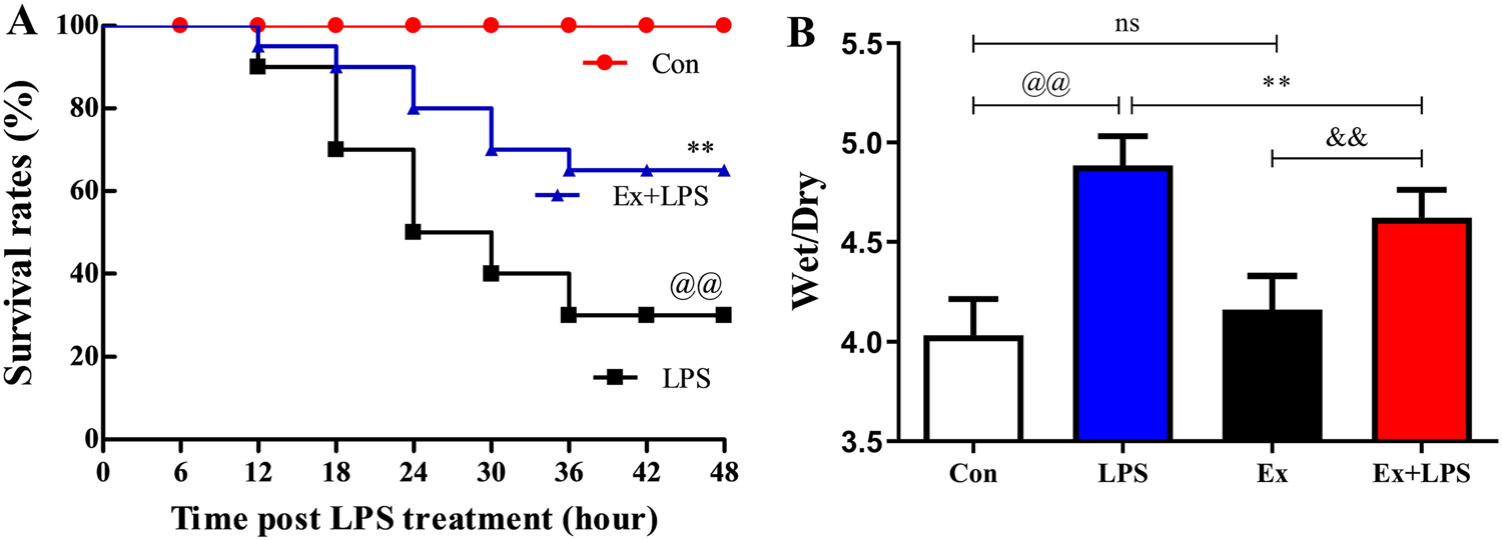
4 weeks of exercise pretreatment improved mortality and W/D. (**A**) Survival rate analysis, (**B**) Detection of W/D in lung tissue. ^@^*P* < 0.05, Con versus LPS groups; *P < 0.05, LPS versus Ex + LPS groups; ^&^*P* < 0.05, Ex versus Ex + LPS groups. *W/D* wet weight/dry weight ratio.

Sham-operated mice exhibited 100% survival. Compared with untrained mice, trained mice showed dramatically improved survival during sepsis (*P* < 0.01; Fig. 10A).

### AE relieved pulmonary edema

Compared with the Con group, the W/D of lung tissues increased significantly after LPS injection (*P* < 0.001). AE prevented the upregulation of W/D in lung tissues (*P* < 0.01; Fig. 10B).

## Discussion

Previous studies have demonstrated that the Warburg effect was involved in innate and adaptive immunity^21^. However, it was not clear whether AE can regulate the Warburg effect during sepsis. There was convincing evidence that increased serum lactate levels were a biomarker of mortality and MODS during sepsis and lactate clearance was a novel therapeutic strategy for sepsis^23–25^. It was well documented that HMGB1 was involved in the development of sepsis and was a promising therapeutic target for sepsis treatment^26,27^. Increased aerobic glycolysis released a lot of lactate which stimulated macrophages to release HMGB1^28^. Our data demonstrated that LPS injection prominently upregulated the levels of lactate and HMGB1 in serum, while AE prominently downregulated the levels of lactate and HMGB1 in serum during sepsis. It has been suggested that insulin can decrease serum HMGB1 levels in septic animals^1^, implying a possible role of glucose metabolism in the regulation of HMGB1 release. It’s well documented that exercise can improve insulin resistance. We came up with a hypothesis that exercise may serum insulin concentrations during sepsis. Our data demonstrated that exercise prominently increased serum insulin concentrations during sepsis. Based on these results, AE improved sepsis via impairing LPS-induced HMGB1 release. AE impaired HMGB1 release via decreasing lactate and Glut1 expression and increasing insulin expression during sepsis. Our research found a new mechanism through which AE improved sepsis.

Increased aerobic glycolysis consumed a large amount of glucose. Strikingly, our data demonstrated that LPS administration markedly decreased blood glucose levels. However, a 4-week exercise pretreatment markedly increased blood glucose levels. Some studies found that LPS administration resulted in hyperglycemia^37,38^. However, other studies found that LPS administration resulted in hypoglycemia^39–41^. Our data demonstrated that LPS administration resulted in hypoglycemia, in part because increased aerobic glycolysis consumed a large amount of blood glucose during sepsis. Hypoglycemia was a common complication of sepsis. If sepsis-induced hypoglycemia was not treated promptly, patients can deteriorate further and fall into a coma, which was easily confused with coma caused by infection, leading to delayed diagnosis and treatment or even death. Hence, septicemia patients should be monitored, and the blood glucose should be supplemented in a timely manner. Our data showed that AE prevented the decrease in blood glucose levels. Our data demonstrated that AE can be used as a preventive tool for LPS-induced hypoglycemia.

Our data revealed that a 4-week exercise pretreatment reduced sepsis-associated lung, liver, kidney, and heart injury. Our data were consistent with previous conclusions^15–19^. Strikingly, we found that AE prevented sepsis-induced aortic injury. The aortic injury occurred before the onset of inflammatory infiltration and organ injury. Previous studies have overlooked the effects of sepsis on the aorta. Aorta could regulate arterial blood pressure and hypotension was one of the most frequent complications of sepsis. Sepsis-induced hypotension led to organ dysfunction and septic shock, which were the most severe complications of sepsis and deadly disease^20^. Our data identified that LPS administration increased aortic media thickness and reduced the area ratio of elastic fibers, which was improved by a 4-week AE pretreatment. These results demonstrated that AE could be used as a preventive tool for LPS-induced aortic injury.

Previous researches demonstrated that neutrophils were involved in the development of sepsis^42^. Previous work identified that deleterious accumulation of neutrophils in organs resulted in MODS^43^. Our data showed that LPS injection resulted in severe neutrophil infiltration in lung, liver, kidney, and heart tissues. Strikingly, our data showed that AE reduced neutrophil infiltration in lung, liver, kidney, and heart tissues during sepsis. Our data identified that deleterious activation of neutrophils was a critical reason leading to host tissue injury and organ damage during sepsis and AE improved sepsis-induced MODS in part by decreasing neutrophil content. There was convincing evidence that TNF-α, CXCL-1, and CXCL-8 were chemotactic for neutrophils. Our data demonstrated that AE prominently reduced BALF levels of CXCL-1, CXCL-8, and TNF-α and CXCL-1, CXCL-8, and TNF-α mRNA expression levels in lung tissue. Hence, AE inhibited neutrophil infiltration via suppressing TNF-α, CXCL-1, and CXCL-8 expression.

Our data demonstrated that LPS injection led to an excessive inflammatory response, pulmonary edema, and the infiltration of inflammatory cells, which were the three main features of ALI^6^. A 4-week exercise pretreatment improved the degree of pulmonary edema and neutrophil infiltration. Hence, AE could be used as a preventive tool for sepsis-associated ALI.

Oxidant/antioxidant imbalance was involved in the pathogenesis of sepsis and LPS administration led to oxidative stress injury^5^. We demonstrated the antioxidant effects of exercise during sepsis. Our data identified that AE prominently increased MDA and MPO expression and prominently decreased SOD and GSH expression during sepsis. Based on these results, AE was a preventive tool for sepsis partly because AE increased antioxidant capacity in lung tissue.

Sirt-1 exerted the effects of promoting lung cell proliferation and vitality and exerted immunomodulatory effects and modulated redox balance^44,45^. Activation of Sirt-1 by agents including resveratrol improved sepsis because Sirt-1 reduced inflammatory response and modulated redox balance^46,47^. Sirt-1 was known to protect against sepsis through Sirt-1/Nrf-2 signaling^47^. Previous studies showed that exercise activated Sirt-1 in muscle tissue because exercise increased NAD/NADH ratio^48^. Sirt-1/Nrf-2 signaling was established as a crucial mechanism underlying lung protection, our data identified that AE could activate Sirt-1/Nrf-2 signaling and reduced inflammatory response and modulated redox balance. Sirt-1/Nrf-2 signaling was a novel therapeutic strategy for sepsis. In contrast to traditional therapeutic methods, AE was a comprehensive intervention treatment. AE improved sepsis through multiple mechanisms simultaneously. The protective effects of 4 weeks of exercise pretreatment on LPS-induced changes MODS, aortic injury, neutrophilic inflammation, pulmonary inflammation, and oxidative stress injury were detected. All of these factors cooperated to improve the survival rate of septic mice.

### Perspective

In conclusion, our results showed that physical exercise can be used as a preventive tool for sepsis-associated death, hypoglycemia, MODS, and aortic injury partly because AE regulated the Warburg effect. AE, which impaired HMGB1 release during sepsis, was a new therapeutic strategy targeting aerobic glycolysis for sepsis. AE impaired HMGB1 release via decreasing lactate and Glut 1 expression and increasing insulin expression during sepsis. AE improved lung injury by alleviating neutrophilic inflammation, oxidative stress injury as well as activating Sirt-1/Nrf-2 signaling. AE inhibited neutrophil infiltration in part by suppressing TNF-α, CXCL-1, and CXCL-8 expression. Our work was still not deep enough, further studies will be needed to identify the underlying mechanism of AE on aortic injury, Warburg effect, and glucose homeostasis during sepsis.

## Supplementary Information

Supplementary Information 1.

Supplementary Information 2.

## Data Availability

The datasets used or analyzed in the current study were available from the corresponding author TD on reasonable request.

## Abbreviations

AE: Aerobic exercise
ALI: Acute lung injury
BG: Blood glucose
BALF: Bronchoalveolar lavage fluid
CXCL: C-X-C motif chemokine ligand
HMGB1: High Mobility Group Box 1
LPS: Lipopolysaccharide
MDA: Malondialdehyde
MODS: Multiple organ dysfunction syndromes
MPO: Myeloperoxidase
Nrf-2: Nuclear factor erythroid 2-related factor 2
Glu1: Glucose transporter 1
GSH: Glutathione
Sirt-1: Silent information regulator 1
SOD: Superoxide dismutase
IL: Interleukin
TNF: Tumor necrosis factor
PFA: Paraformaldehyde
W/D: Wet weight/dry weight ratio
