# Author Correction: Aerobic exercise improves LPS-induced sepsis via regulating the Warburg effect in mice

**DOI:** 10.1038/s41598-022-09661-4

**Published:** 2022-04-01

**Authors:** Xishuai Wang, Zhiqing Wang, Donghui Tang

**Affiliations:** 1 Department of College of P.E and Sport, Beijing Normal University, No. 19, Xinjiekouwai St, Haidian District, Beijing, 100875 People’s Republic of China; 2 Department of Animal Genetic Resources, Institute of Animal Science, Chinese Academy of Agricultural Sciences, Beijing, 100193 People’s Republic of China

Correction to: *Scientific Reports* https://doi.org/10.1038/s41598-021-97101-0, published online 07 September 2021

The original version of this Article contained an error in Figure 4, where the incorrect images for panels A and B were submitted for publication. The original Figure 4 and accompanying legend appear below.

The original Article has been corrected.

**Figure 4:**
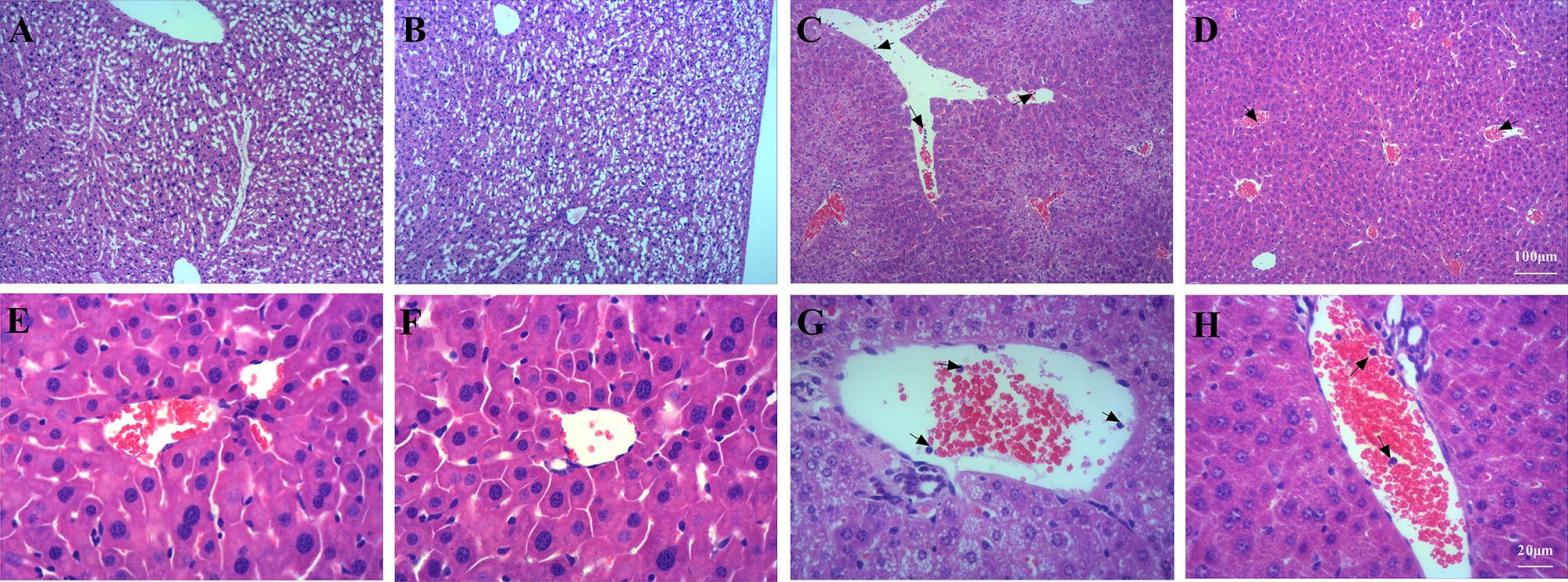
A photomicrograph of liver tissues used for morphological analysis. (**A**) Con group, (**B**) LPS group, (**C**) Ex group, (**D**) Ex + LPS group. White arrows indicate neutrophils [(**A**–**D)** × 100 magnification; (**E**–**H**) × 400 magnification].

