# Supplementary material for: Aerobic exercise improves LPS-induced sepsis via regulating the Warburg effect in mice
Source: Sci Rep. 2021 Sep 7;11:17772. doi: 10.1038/s41598-021-97101-0 (PMC8423727; doi:10.1038/s41598-021-97101-0)
Supplement: Supplementary file 1 — Supplementary Information 1. [file 41598_2021_97101_MOESM1_ESM.docx]

**Supplementary Materia 1**

Statistical methods were presented in Supplementary Material 1. Neutrophil content, lung injury score, kidney injury score, liver injury score, pulmonary fibrosis score were measured, myocardial injury score, and aortic intima media-thickness were measured.

**Neutrophil content:** By using a 100-point grid of a known area (62,500 μm^2^ at 400X magnification) that was attached to the ocular of the microscope, we counted the number of points that were hitting the tissue samples and the numbers of neutrophils and positive cells in each field. The cell density was determined as the number of positive cells in each field divided by the tissue area, and this value was expressed as cells/mm^2^ [1]. Morphometric measurements were performed in 15 fields for each animal at 400X magnification by an investigator who was blinded to the specific group that was studied.

**Kidney injury score:** Acute tubular damage was assessed via hematoxylin and eosin (HE) staining by using previously described criteria [2]. Acute tubular damage was scored by using a semiquantitative scoring system (0 to 3 points) for each criterion in 20 randomly sampled high-power fields of the cortex per animal (0 = absent, 1 = mild, 2 = moderate, and 3 = strong acute tubular damage).

**Liver injury score:** Formalin-fixed liver tissues were embedded in paraffin wax, serially sectioned, and then stained with HE. Morphological characteristics (including PMN infiltration, interstitial edema, focal necrosis, and hemorrhage/congestion) were evaluated under a light microscope, and Suzuki scores (i.e., the sum of the scores of congestion [0: none; 4: severe], vacuolization [0: none, 4: severe], and necrosis [0: none; 4: > 60%]) were calculated to determine the liver injury level [3,4].

**Lung injury score:** The lung tissues were exposed to paraffin procedures and sectioned at approximately 5 μm thick, after which they were stained with HE, as has been previously described. Lung injury was evaluated and scored by two pathologists who were blinded to the experimental design by using a recent criterion [5], in which lung damage was evaluated on a two-point scale with scores ranging from 0 to 1.

**Myocardial injury score:** Cardiac sections were prepared as previously described and stained with HE according to standard procedures. A pathologist was assigned to grade the MI injuries in a blinded fashion. Histological analyses of infarct size, hemorrhage, and leukocyte infiltration were scored as being none, weak, moderate, strong, or very strong (scores of 0, 1, 2, 3, or 4, respectively). This method for the objective quantification of MI injury has been previously described [6-8].

**Aortic intima media-thickness:**

Ascending thoracic aorta was dissected and rinsed with cold phosphate-buffered saline and placed in 10% neutral buffered formalin until sectioning for microscopic analysis. Aortic specimens were serially sectioned into three or four rings and embedded entirely in tissue blocks for hematoxylin and eosin (HE) staining. Histologic sections were then reviewed by one person (O.T.) and scanned by a computer assisted imaging device (CAS-200 Cell Analysis System; Bacus Laboratory, Chicago, IL, USA) for measurement of the vessel wall thickness. The mean value of the vessel wall thickness from the endothelial surface to the adventitia was recorded from 10 different locations spanning the entire crosssection. The mean values of aortic wall thickness were first normalized for overall body weight, since it has been shown that larger mice generally exhibit larger vessel wall thickness [9].

**The density of neutrophils in liver, kidney, and heart tissues (cells/mm^2^).**

|  | **Con** | **Ex** | **LPS** | **Ex + LPS** | **P** |
| --- | --- | --- | --- | --- | --- |
| The density of neutrophils in liver tissue | 41.54 ± 8.65 | 37.93 ± 5.07 | 655.32 ± 113.22^@@@^ | 541.47 ± 58.56^**^ | < 0.01 |
| The density of neutrophils in kidney tissue | 53.11 ± 8.96 | 45.86 ± 8.08 | 587.74 ± 103.64^@@@^ | 481.63 ± 52.99^**^ | < 0.01 |
| The density of neutrophils in heart tissue | 38.54 ± 6.47 | 32.72 ± 6.04 | 379.78 ± 59.62^@@@^ | 227.81 ± 24.46^**^ | < 0.01 |

LPS injection increased neutrophil content in liver, kidney, and heart tissues (*P* < 0.01), while exercise attenuated neutrophil content in liver, kidney, and heart tissues (*P* < 0.01). @@@, *P* < 0.001, the difference is significant between the Con and LPS groups. **, *P* < 0.01, the difference is significant between the LPS and Ex + LPS groups. Values are expressed as the means ± SD.

**Detection of organ injury.**

|  | **Con** | **Ex** | **LPS** | **Ex + LPS** | **P** |
| --- | --- | --- | --- | --- | --- |
| Liver injury score | 0.15 ± 0.02 | 0.13 ± 0.01 | 6.78 ± 0.71^@@@^ | 3.51 ± 0.48^***^ | < 0.01 |
| Kidney injury score | 0.52 ± 0.06 | 0.43 ± 0.03 | 6.54 ± 0.89^@@@^ | 3.25 ± 0.72^***^ | < 0.01 |
| Lung injury score | 2.16 ± 0.29 | 2.44 ± 0.27 | 7.45 ± 0.87^@@@^ | 4.52 ± 0.63^***^ | < 0.01 |
| Myocardial injury score | 0.13 ± 0.01 | 0.18 ± 0.01 | 2.79 ± 0.27^@@@^ | 2.16 ± 0.17^*^ | < 0.01 |
| Aortic intima media-thickness (mm) | 51.4 ± 10.12 | 50.46 ± 15.6 | 65.46 ± 11.8^@@^ | 57.46 ± 11.8^*^ | < 0.01 |
| Aortic-intima media thickness (mm/g of body weight) | 1.89 ± 0.24 | 1.91 ± 0.25 | 2.3 ± 0.22^@@^ | 2.1 ± 0.19^*^ | < 0.01 |

Liver injury score, kidney injury score lung injury score, myocardial injury score, aortic intima media-thickness were measured. @@, *P* < 0.01, the difference is significant between the Con and LPS groups. @@@, *P* < 0.001, the difference is significant between the Con and LPS groups. *, *P* < 0.05, the difference is significant between the LPS and Ex + LPS groups. **, *P* < 0.01, the difference is significant between the LPS and Ex + LPS groups. Values are expressed as the means ± SD.

**Reference**

[1] Lanças T, Kasahara DI, Prado CM, et al. Comparison of early and late responses to antigen of sensitized guinea pig parenchymal lung strips. J Appl Physiol. 2006;100:1610-1616. doi: 10.1152/ japplphysiol.00828.2005.

[2] Bockmeyer CL, Reuken PA, Simon TP, et al. ADAMTS13 activity is decreased in a septic porcine model. Significance for glomerular thrombus deposition. Thromb Haemost. 2011;105:145-153. doi: 10.1160/TH10-03-0153.

[3] Cay-Huyen Chen, Pei-Shan Tsai, Chun-Jen Huang. Minocycline ameliorates lung and liver dysfunction in a rodent model of hemorrhagic shock/resuscitation plus abdominal compartment syndrome. J Surg Res. 2013;180(2):301-9. doi: 10.1016/j. jss.2012.04.036.

[4] S Suzuki, S Nakamura, T Koizumi, et al. The beneficial effect of a prostaglandin I2 analog on ischemic rat liver. Transplantation. 1991;52(6):979-83. doi: 10.1097/ 00007890-199112000-00008.

[5] Gustavo Matute-Bello, Gregory Downey, Bethany B Moore, et al. An official American Thoracic Society workshop report: features and measurements of experimental acute lung injury in animals. Am J Respir Cell Mol Biol. 2011;44(5):725-38. doi: 10.1165/rcmb.2009-0210ST.

[6] Paul-Mihai Boarescu , Ioana Chirilă, Adriana E Bulboacă, et al. Effects of Curcumin Nanoparticles in Isoproterenol-Induced Myocardial Infarction. Oxid Med Cell Longev. 2019;2019:7847142. doi: 10.1155/2019/7847142.

[7] Erman Caner Bulut, Leyla Abueid, Feriha Ercan, et al. Treatment with oestrogen-receptor agonists or oxytocin in conjunction with exercise protects against myocardial infarction in ovariectomized rats. Exp Physiol. 2016;101(5):612-27. doi: 10.1113/EP085708.

[8] Mohamed Abdellah Ibrahim, Ayman Geddawy, Soha Abdel-Wahab, et al. Sitagliptin prevents isoproterenol-induced myocardial infarction in rats by modulating nitric oxide synthase enzymes. Eur J Pharmacol. 2018;829:63-69. doi: 10.1016/ j.ejphar. 2018.04.005.

[9] Kottarappat N Dileepan, Thomas P Johnston, Yuai Li, et al. Deranged aortic intima-media thickness, plasma triglycerides and granulopoiesis in Sl/Sl(d) mice. 2004;13(5-6):335-41. doi: 10.1080/09629350400008794.
