# Supplementary material for: Aerobic exercise improves LPS-induced sepsis via regulating the Warburg effect in mice
Source: Sci Rep. 2021 Sep 7;11:17772. doi: 10.1038/s41598-021-97101-0 (PMC8423727; doi:10.1038/s41598-021-97101-0)
Supplement: Supplementary file 2 — Supplementary Information 2. [file 41598_2021_97101_MOESM2_ESM.docx]

**Supplementary Materia 2. PCR** **primer sequence information of mouse.**

| Gene name | Primer sequence |
| --- | --- |
| CXCL-1 | CTGGGATTCACCTCAAGAACATC |
|  | CAGGGTCAAGGCAAGCCTC |
| CXCL-8 | ATGCCCTCTATTCTGCCAGAT |
|  | GTGCTCCGGTTGTATAAGATGAC |
| IL-1RN | GCTCATTGCTGGGTACTTACAA |
|  | CCAGACTTGGCACAAGACAGG |
| IL-6 | CCAAGAGGTGAGTGCTTCCC |
|  | CTGTTGTTCAGACTCTCTCCCT |
| 1L-10 | GCTCTTACTGACTGGCATGAG |
|  | CGCAGCTCTAGGAGCATGTG |
| TNF-α | ATGTCTCAGCCTCTTCTCATTC |
|  | GCTTGTCACTCGAATTTTGAGA |
| Sirt-1 | GCTGACGACTTCGACGACG |
|  | TCGGTCAACAGGAGGTTGTCT |
| Nrf-2 | TCTTGGAGTAAGTCGAGAAGTGT |
|  | GTTGAAACTGAGCGAAAAAGGC |
| GAPDH | AGGTCGGTGTGAACGGATTTG |
|  | TGTAGACCATGTAGTTGAGGTCA |
